# Adjusting the scope of natural killer cells in cancer therapy

**DOI:** 10.1038/s41423-025-01297-4

**Published:** 2025-05-23

**Authors:** Zihen Shen, Xiangpeng Meng, Jai Rautela, Michael Chopin, Nicholas D. Huntington

**Affiliations:** 1 Biomedicine Discovery Institute and the Department of Biochemistry and Molecular Biology, Monash University, Clayton, VIC Australia; 2 oNKo-Innate Pty Ltd., Moonee Ponds, VIC Australia

**Keywords:** Natural Killer cell, Immunotherapy, Innate Immune cells, Cytokines, Drug Development, Cancer, Immunosurveillance, Cytokines, Target validation, Immunotherapy

## Abstract

Natural killer (NK) cells have evolved to detect abnormalities in tissues arising from infection with pathogens, genomic damage, or transformation and respond rapidly to the production of potent proinflammatory and cytolytic mediators. While this acute proinflammatory response is highly efficient at orchestrating sterilizing immunity to pathogens in a matter of days, cellular transformation often avoids the innate detection mechanisms of NK cells. When cellular transformation results in malignancy, tumor cells and/or the tumor microenvironment can evolve additional mechanisms to circumvent NK cell responses, and cancer is now a dominant disease burden worldwide. Here, we review recent advances in our understanding of the combined relationship between malignancies and natural killer (NK) cells, learn from recent clinical efforts in therapeutically targeting natural killer (NK) cells in cancer and outline some emerging therapeutic concepts that aim to improve the innate immune response against cancer.

## NK cell-targeted immunotherapies

Over the past decade, we have continued to witness the importance of adaptive, T-cell–mediated immunity to cancer. The class of drugs that specifically target immune cell biology (known as immunotherapies) has achieved US Federal Drug Administration (FDA) approval in more than 20 cancer types (https://www.cancerresearch.org). Antibodies against PD1, LAG3 and CTLA-4, which block major inhibitory receptors on T cells, are among the most commonly used drugs for advanced solid cancer treatment [1], with Merck’s Keytruda (pembrolizumab; anti-PD1) leading to the highest net sales of all oncology drugs on the market this year. More recently, tumor immune lymphocyte (TIL) therapy was also approved for the treatment of metastatic melanoma. Here, a biopsy of a patient’s melanoma is harvested, and T cells are isolated, expanded and reinfused back into the patient to increase the frequency of effector T cells that can directly recognize melanoma neoantigens [2]. There are also encouraging data suggesting that vaccines encoding melanoma neoantigens can achieve the same effects [3]. In hematological malignancies, genetically engineered T cells that express chimeric antigen receptors (CAR) against tumor-restricted antigens and bispecific antibodies that simultaneously bind T cells and tumor cells, forcing their interactions and T-cell activation, result in robust and durable responses [4]. In contrast, positive data from therapeutic trials designed to increase the NK cell frequency and antitumor activity of cancer patients have yet to emerge. Many of the approaches to harnessing NK cell activity in cancer patients are directly based on the successful approaches that target patient T cells highlighted above. However, T-cell biology has evolved significantly from that of their innate ancestors, and much of the T-cell effector biology that underlies these therapeutic responses is either smaller in magnitude in NK cells or qualitatively different. Thus, the NK cell cancer immunotherapy field must learn from these early clinical shots-on-goal and will respond with more nuanced and differentiated approaches to harness NK cell effector responses in safer, more targeted manners, resulting in clinical activity in advanced cancer patients who need them most.

### NK cell biology in cancer: opportunities for orthogonal immunotherapies

To better equip ourselves with NK cell-targeting approaches, it is an opportune time to reflect on the key biological functions of NK cells during an antitumor immune response. The NK cell‒cancer immunity cycle was recently outlined in detail, documenting the major interactions between NK cells and tumor cells and how these interactions both shape the tumor microenvironment and T-cell responses to immunotherapy and control large established tumors [5]. Briefly, NK cell activation status can be reduced to the net outcome of multiple stimulatory inputs (from activating receptors and inflammatory cytokines) and suppressive inputs (from inhibitory receptors and immunosuppressive factors). In healthy human adults, a large pool of mature NK cells circulate in the blood. These NK cells contain cytotoxic granules and can rapidly produce type I proinflammatory cytokines and chemokines; however, they do not react against healthy self-tissues because of the presence of stably expressed inhibitory ligands (e.g., MHC-I) on all healthy tissues and the lack of or very low expression of stimulatory ligands on healthy tissues (e.g., ULBP1) [5]. Furthermore, inflammatory cytokines are minimally expressed in healthy adults; thus, the overall immune activation status of healthy NK cells can be considered poised or ‘armed, but not dangerous’, analogous to having a loaded gun, but the safety latch is ON. This is the advantage of NK cells in driving rapid inflammation in response to pathogen infection because their status can very quickly change when multiple stimulatory inputs are added in response to pathogen recognition.

### Releasing the NK cell ‘Safety Switch’

The dominant inputs that tip NK cell activation from ‘poised’ into raging effector cells are pro-inflammatory cytokines produced by their innate immune counterparts, including dendritic cells and macrophages. Potent type-I cytokines such as IL-18 and IL-12 are readily produced from tissue macrophages and dendritic cells at sites of inflammation and are stimulated via pathogen-associated molecular patterns (PAMPs) and damage-associated molecular patterns (DAMPs) [6]. IL-12 acts locally on mature NK cells that constitutively express IL-12RB1/2 to rapidly induce local IFNγ production, with both immune-intrinsic and immune-extrinsic (acting on IFNγR^+^ infected/tumor tissues) IFNγ activity contributing to sterilizing immunity [6]. An additional input that balances NK cell activation with ‘ON’ is the induction of stress ligands on DNA-damaged, stressed, infected host cells. Stress ligands are germline-encoded ligands that are not expressed on healthy cells; however, they are induced following immunogenic cell death, pathogen infection and cellular transformation [7]. These ligands are sensed by activating receptors such as NKG2D, NKp30 and NKp46 and focus NK cell effector responses against pathogenic cells, including cell lysis via granzymes and perforin production [5]. Thus, mature NK cells can rapidly drive targeted and nontargeted inflammatory responses that act as catalysts for adaptive responses against pathogens. An example of this is local IFNγ production, which can upregulate MHC-I on IFNγR^+^ pathogenic cells and professional antigen-presenting cells such as cDC1s for optimal CD8+ T-cell priming and antigen-specific cytotoxic effector responses [6, 8]. How these NK cell activation cues are induced in cancer and by which cell types and their kinetics during tumorigenesis are still being resolved. Equally, the earliest immune cells to detect cancerous cells/altered self-tissues to initiate tumor-immune infiltration and disease control is still being debated. NK cells are very efficient at detecting and eradicating tumor cells outside of the tumor microenvironment; for example, in experimental models of metastases, it is highly plausible that NK cells perform this innate immune surveillance function constantly during healthy adulthood [9]. Indeed, the inverse correlation between NK cell activity and cancer incidence supports a role for NK cells in human cancer immunosurveillance [10]. Furthermore, patients with high “NK cell signature” scores in solid cancers also have significantly longer periods of disease-free survival than patients with low “NK cell signature” scores in an identical solid cancer setting [11, 12].

### Single Combat. NK cells versus cancer metastases

Thus, NK cells likely contribute to antimetastatic immunity by detecting single or small clusters of metastatic cancer cells that have recently entered blood or lymph vessels via intravasation and killing them before they can extravasate into organs and form micrometastases. Recent data from pancreatic ductal cell adenocarcinoma suggest that circulating tumor cells exploit the CD94/NKG2A inhibitory axis via the expression of HLA-E to avoid peripheral NK cell lysis [13]. Clustering of cancer cells in circulation has been identified across various tumor types, such as breast, lung, prostate, kidney and melanoma, and circulating tumor cell clusters metastasize 20–100 times more efficiently than single circulating tumor cells do and are associated with poorer overall survival outcomes [14]. Thus, clustering must efficiently shield some circulating tumor cells from NK cell immunosurveillance long enough for the extravasation process to occur. This shielding needs to occur only briefly, as the window for NK cell detection and killing of metastatic cells is short. Using highly sensitive bioluminescence imaging and intravital two-photon microscopy, NK cells were shown to eliminate metastases from the lung within 24 h of arrival but not thereafter. Approximately half of the NK cell‒metastasis interactions lead to rapid tumor cell death within 4 h of tumor cell seeding; however, 24 h after seeding, nearly 100% of these NK cell‒metastasis interactions result in survival of the metastases [15]. The caveat here is that these studies model experimental metastases where a bolus of single tumor cells are introduced intravenously at once; however, in the context of solid metastatic tumors, single spontaneous metastases are likely to be continually entering the circulation and constantly subjected to NK cell immunosurveillance. Indeed, several preclinical models of spontaneous metastasis exist, including mammary fat pad orthotopic breast cancer models where spontaneous metastases are detected at peripheral sites, predominantly in the lungs. Recently, using a model involving transplantation and surgical resection of the murine MHC-I^+^Rae1b^+^ breast cancer line, EO771 showed that administering IL-15/IL-12-conditioned syngeneic NK cells following primary tumor removal enhanced the long-term survival of mice with minimal metastatic spread [16].

### Calling for backup

These data suggest that metabolically fit and highly functional NK cells can continually contribute to metastatic immunity after the primary tumor and continuing source of metastases are removed. Interestingly, the therapeutic impact of adoptively transferred NK cells was dependent on endogenous CD8 + T cells with NK cell adoptive therapy facilitating the development of a tumor-specific CD8^+^ T-cell response in the host. This therapy generated memory CD8 + T cells that protected against tumor rechallenge and prevented tumor formation when transferred into naive mice. Mechanistically, transferred NK cells resulted in the accumulation of DCs within the metastatic lung, and IFNγ derived from these NK cells was key to optimal DC activation, as evidenced by the upregulation of CD86, CD40, and MHCI/II. Furthermore, the production of IFNγ by NK cells is required for the accumulation of activated CD8^+^ effector T cells and precursors of exhausted CD8^+^ T cells (Tpex) within the metastatic lung. Building upon these preclinical insights, this group conducted an investigator-initiated clinical trial using a similar protocol in a small number of cancer patients. Preliminary findings revealed that post-resection treatment with IL-15/12-conditioned autologous NK cells in patients with diverse solid metastatic malignancies was well tolerated and displayed some early signs of efficacy. These results provide evidence that autologous NK cell therapy can positively impact the disease control of established low-burden MHC-I^+^ tumor metastases by both directly detecting and killing circulating metastatic tumor cells (*Single Combat*) and by leading to improved immune infiltration and cDC1 and CD8^+^ T-cell activation and priming (*calling for backup*) to establish antigen-specific control of newly established metastases.

### NK cells as master strategists for solid tumor battlefields

While many studies on NK cells over the past 20 years have focused on their role in controlling circulating tumor cells, e.g., hematological malignancies or metastases, emerging data suggest that NK cells also play important roles in solid tumor immunity [5]. Activated NK cells are specialized producers of several chemokines required for pro-inflammatory immune cell chemotaxis, namely, XCL1 and CCL3/4/5. Several recent studies have correlated tumor-resident NK cell abundance and the production of these chemokines with tumor-immune infiltration and tumor immunity [17, 18]. NK cell-derived XCL1 has been strongly implicated in the direct recruitment and activation of Clec9a^+^XCR1^+^ cDC1s into solid tumors, with cDC1s being essential for tumor antigen cross-presentation in tumor-draining lymph nodes [17]. The importance of this NK cell–cDC1 axis has been well demonstrated in tumor models lacking the *Ptgs1* and *Ptgs2* genes, which are required to produce the immunosuppressive prostaglandin PGE2. The inability of tumor lines to produce PGE2 renders them highly susceptible to cDC1-dependent CD8^+^ T-cell-mediated immune control [19], and researchers have exploited preclinical *Ptgs1/Ptgs2*^-/-^ tumor models to characterize the mechanisms controlling cDC1 accumulation. PGE2 appears to be a dominant NK cell immunosuppressant, downregulating several activating receptors on human NK cells, including NKG2D and NKp30, and suppressing the frequency of NK cells and the production of the chemokines XCL1 and CCL5 in preclinical models [20, 21]. *Ptgs1/Ptgs2*^-/-^ tumor models have implicated tumor-resident NK cells as important sources of the chemokines required for optimal cDC1 recruitment into solid murine tumors, and similar correlative data from human solid cancers, such as melanoma, breast cancer, and lung cancer, also support this NK cell/XCL1 axis as a driver of cDC1 infiltration [17].

**Fig. 1:**
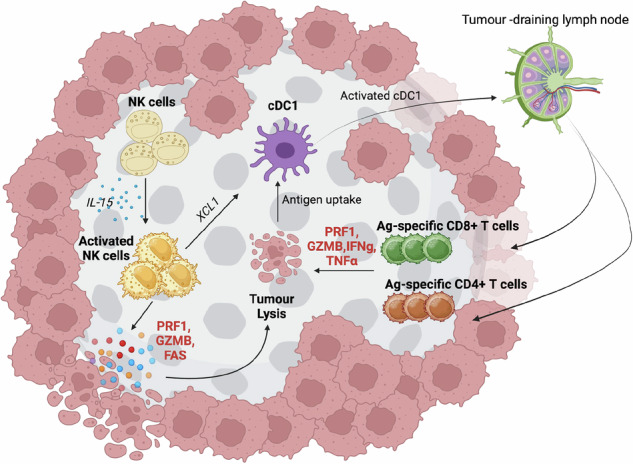
NK cells promote solid tumor inflammation. NK cells within the tumor microenvironment can drive local inflammation when activated appropriately. NK cells lyse tumor cells, generating tumor antigens for uptake and cross-presentation to T cells via cDC1s in tumor-draining lymph nodes. Activated NK cells produce proinflammatory chemokines and cytokines that directly impair tumor growth, attract additional antitumor effector cells, increase tumor antigen/MHC expression, and increase the frequency and function of tumor-specific T cells

While XCR1, the receptor for XCL1, is highly restricted to cDC1s, many different immune cells, including monocytes and effector T cells, express receptors (CCR1/3/4/5) for the other NK cell-derived chemokines CCL3/4/5. A role for NK cell-derived CCL5 in recruiting T cells into solid tumors was previously shown via a genetically engineered mouse model of lung adenocarcinoma (Lenti-Cre KRAS G12D^+^ P53-null model) [22]. In this model, late-stage tumors exhibited poor lymphocyte infiltration, infrequent NK cells and exhausted tumor-resident T-cell populations. To investigate whether endogenous NK cell responses could be bolstered in tumor-bearing animals, temporal expression of an NK cell activation ligand, m157 (derived from murine cytomegalovirus, which binds to activating Ly49H on NK cells), on tumor cells was employed. Inducible expression of m157 on lung tumor cells results in a transient increase in NK cell activity and abundance, with CCL5 expression being upregulated in Ly49H^+^ NK cells and leading to increased CD8 + T-cell recruitment and activation within tumors. While this model relies on the transgenic induction of a viral ligand to stimulate tumor-resident NK cells, we recently exploited a rationally designed small-molecule inhibitor combination to induce a similar phenomenon [23]. Germline-encoded NKG2D ligands are commonly upregulated on tumor cells following DNA damage and cellular senescence, which can induce NK cell activation via NKG2D, a ubiquitously expressed activating receptor on NK cells. The activation of wild-type p53 in tumor cells induces apoptosis and NKG2D ligand expression, which contributes to immunogenic cell death by activating local NK cells. It was hypothesized that targeting the p53 pathway in cancer cells could transiently upregulate NKG2D ligands on tumor cells, thus increasing tumor immunogenicity to NK cells, igniting a proinflammatory tumor microenvironment, and ultimately enhancing CD8^+^ T-cell control of preclinical solid tumors [24]. Previous work has indicated that inhibiting WEE1 induces cell cycle arrest through the induction of DNA damage and the transcriptional activity of p53 in melanoma cells, whereas AKT was shown to mediate the ubiquitin-mediated degradation of p53 in melanoma cells [25]. Moreover, inhibiting AKT/WEE1 signaling via small molecule inhibitors strongly synergistically suppressed melanoma cell growth in vitro and induced the expression of cell membrane calreticulin and NKG2D ligands, two indicators of immunogenic cell death. Using orthotopic preclinical models of anti-PD1 resistant, immune ‘cold’ melanoma (B16F10), the pharmacologic inhibition of AKT and WEE1 via AZD5363 (capivasertib) and MK1775 (adavosertib), respectively, was able to induce robust anti-PD1 responsiveness. Mechanistically, the combination of capivasertib and adavosertib resulted in an inflamed tumor microenvironment characterized by increased expression of markers of immunogenic cell death, including calreticulin, and notable increases in cDC1 and NK cell infiltration. While combination therapy had a clear antitumor effect in this preclinical melanoma model, the addition of anti-PD1 to this combination capivasertib/adavosertib treatment regime resulted in an exceptionally high rate of melanoma cure. As expected, anti-PD1 monotherapy had no effect on B16F10 tumor control, with capivasertib/adavosertib treatment being essential for inducing anti-PD1 responsiveness. The efficacy of triple therapy was found to be dependent on endogenous NK cells, CD8 + T cells and wild-type p53 expression in B16F10 cells with anti-PD1 therapy, further increasing cytotoxic immune cell infiltration and the conversion of the B16F10 tumor microenvironment from ‘cold’ to ‘hot’ characterized by increased expression of CXCL10, CXCL9, CCR5, CCR2, CX3CR1, IL-2, IL-15, PD1 and PDL1. Taken together, effectively targeting tumor-resident NK cell activity promotes tumor inflammation, highlighting the importance of NK cell-derived chemokines in cancer immunity in addition to their well-studied cytolytic functions. These studies demonstrate the value of targeting intrinsic and extrinsic pathways that can enhance tumor immunogenicity to NK cells as an approach to induce anti-PD1 responsiveness and control of CD8 + T cells in immunosuppressed or ‘cold’ tumors if the tumor mutational burden and neoantigen presentation are sufficiently high (Fig. 1).

### NK cell-derived cytokines. A call to arms

In addition to a swath of inflammatory chemokine production, NK cells are specialized producers of proinflammatory and regulatory cytokines. While many of these cytokines, such as IFNγ and TNFα, have direct antipathogen/anticancer effects, NK cell-derived FLT3L appears to be instrumental for the differentiation and maintenance of cDC1s in mice and optimal antitumor activity in preclinical models. In some human cancers, a higher frequency of cDC1s within the TME is associated with better disease outcomes and is proportional to the abundance of FLT3L in the TME [17, 18]. Not surprisingly, given their specialized role in tumor antigen cross-presentation, there was also a clear correlation between cDC1 frequency and anti-PD1 responses in a small melanoma cohort [18]. These findings support the notion that enhancing the frequency and function of NK cells within the TME leads to elevated levels of FLT3LG and, consequently, an increased abundance of cDC1s and neoantigen presentation potential to CD8 T cells in tumor-draining lymph nodes. However, whether the increased abundance of cDC1s is driven by biased differentiation of monocyte precursors within tumors or prolonged cDC1 survival remains to be determined.

Finally, the importance of the impact of NK cell-derived cytokines on tumor-resident immune cell activity is another emerging topic of investigation. Upon activation from tumor ligands, NK cells rapidly produce abundant IFNγ and TNFα in the presence of IL-12/IL-18 derived from activated tumor-resident macrophages and DCs. NK cell-derived IFNγ and TNFα are then able to induce a range of local immune cell activities, including the polarization of helper T cells and cytotoxic T-cell responses [26]. The proinflammatory production of cytokines is obviously important in the TME; however, draining lymph node-resident NK cells and their production of local IFNγ have also been shown to increase the frequency of IFNγ^+^ CD8^+^ T cells being primed by DCs to an experimental antigen [27]. Thus, emerging data suggest that immunotherapy approaches aimed at increasing NK cell fitness/function/abundance should not only reduce metastasis rates and periods of progression-free survival but also positively impact tumor antigen presentation to CD8 + T cells via cDC1s, T-cell Th1 polarization and tumor immune infiltration. Thus, targeting tumor-resident NK cell activity has the potential to overcome certain resistance mechanisms to ICIs mediated by poor neoantigen presentation and has the potential to increase ICI response rates by potentiating CD8^+^ T-cell priming.

## Considerations for Targeting NK Cell Antitumor Immunity

**Fig. 2:**
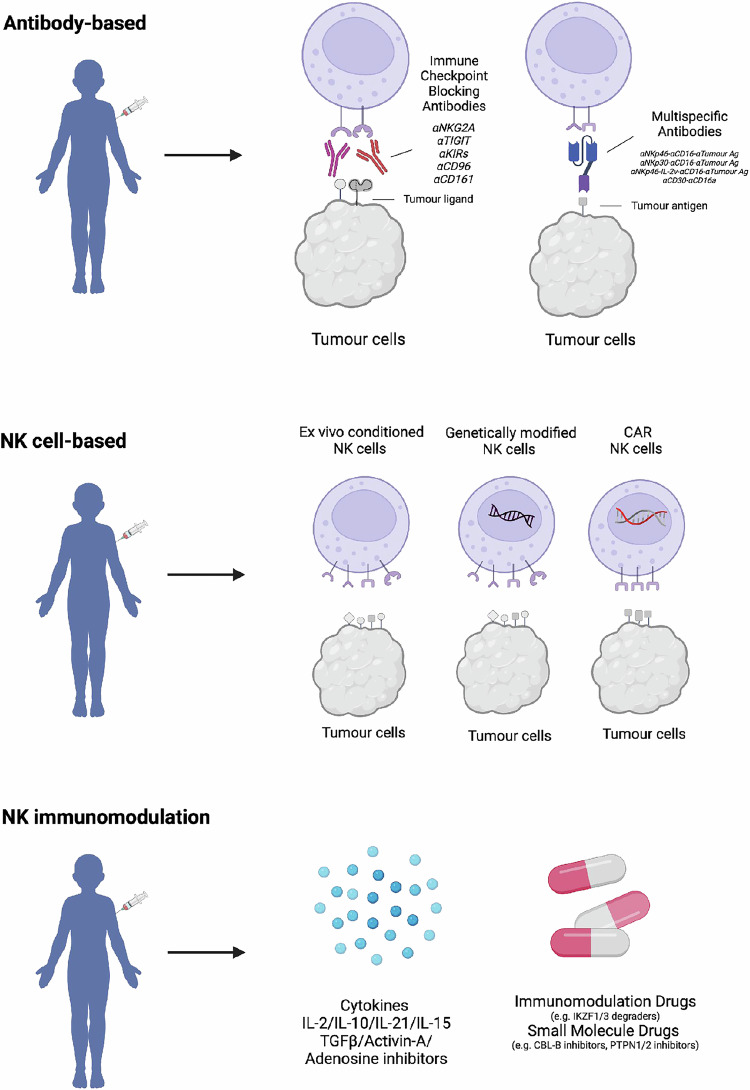
Current trends in NK cell-targeting agents

Given the established role of NK cells in metastatic immunity and their emerging role in driving tumor immune infiltration, therapeutic strategies to increase the frequency and function of endogenous NK cells in cancer patients are predicted to improve patient outcomes. In attempts to exploit NK cell effector functions as either a monotherapy or in combination with orthogonal immunotherapies, chemotherapies and radiotherapies have been pursued since the discovery that NK cells possess spontaneous antitumor and alloreactivity. However, despite the past 40 years of endeavors, there are few examples of clinically validated approaches that enhance NK cell antitumor immunity to achieve durable disease remission. However, the clinical validation of T-cell-biased immunotherapies, including cellular therapies, and the clear beneficial role of NK cells in preclinical cancer models indicate that motivation and belief remain, and many novel approaches and modalities to target NK cells in cancer patients are being pursued in early clinical trials or at the drug discovery/development stage (Fig. 2).

### All the killers are not created equal

There are numerous considerations when strategizing immunotherapy design to best leverage the anticancer activity of NK cells. Questions that one could pose include the following: Can we specifically augment the frequency and function of tumor-resident NK cells? Do we need to? What safety concerns may arise from systemic NK cell activation? Will related innate lymphocytes and invariant T cells also be targeted with this approach, and if so, what are the consequences? What are the dominant NK cell effector levers one wishes to pull? Does repeat NK cell activation compromise NK cell fitness and half-life? Are there enough endogenous NK cells to target or are third-party NK cells required? Does NK cell exhaustion occur in the TME? Is this state distinct from NK cell immunosuppression? Can NK cell exhaustion or immunosuppression be reversed, or must we prioritize preventing these cell states?

Another key consideration for the current crop of NK cell immunotherapies in clinical trials arises from the fact that some of these approaches are based on T-cell-targeted immunotherapy platforms that have resulted in positive clinical data. Therefore, “Are the mechanisms of action that drive this therapeutic response preserved in NK cells or does this exploit biology unique to T cells?” To state this more precisely, using the example of NK cell bispecific/trispecific engagers “are the cellular mechanisms driving the clinical activity when targeting T cells with this modality possible when targeting NK cells in the same manner?”. To explore this example further, the ability of cytotoxic effectors to rapidly synthesize proteins, divide multiple times per day in response to antigen receptor triggering, greatly increasing the number of cytotoxic progeny while recognizing and killing tumor cells are known properties of CD8 T cells [28, 29]; however, it is unclear how efficient these processes are for NK cells within the TME, especially when their innate biological roles are considered. That said, NK cells can rapidly expand and contribute to proinflammatory effector responses over several days when responding to viral infections [30], and the IL-2v moiety in NK cell engager molecules can drive robust NK cell proliferation in vitro and promote the recruitment of healthy, third-party donor NK cells into the TME [31, 32].

### NK cell engagers. The medieval knight with the automatic weapon

Several biotechnology companies are currently developing multispecific NK cell engagers with the aim of emulating the types of clinical responses observed with bispecific T-cell engagers but with potentially improved safety profiles for practical administration and wider clinical use. Multiple specific NK cell engagers have been developed to stimulate NK cells through activating receptors, including NKG2D, CD16, NKp46 and NKp30, whereas T-cell engagers stimulate T cells through the T-cell receptor/coreceptor complex (CD3, CD28, and CD137/41BB). NKG2D, CD16, NKp46 and NKp30 are constitutively expressed on healthy circulating human NK cells, but these receptors can be downregulated when interacting with tumor ligands in cancer patients [31] and are sensitive to cleavage by enzymes in the TME and subsequent activation and by TGF-β [33], as reported in gastric cancer patients [34]. A potential consequence of this is that the baseline expression of the receptors for the NK cell-targeting arm of the engager might be lower than desired, especially in patients with high disease burden. This likely differs from the more stable CD3 expression on polyclonal T cells in the periphery and tumors for T-cell bispecific antibody engagement. Thus, unleashing the full potential of NK cell engagers may require innovative strategies that prevent the downregulation of activating receptors on NK cells. Furthermore, the crosslinking of these receptors in isolation on T cells versus NK cells results in distinct biology. Depending on the extent of receptor crosslinking, CD3/CD28/41BB can induce significant activation and T-cell proliferation, whereas strong crosslinking of NKG2D, NKp46, CD16 or NKp30 induces NK cell activation and degranulation but does not induce NK cell proliferation in isolation because of the strong dependency of NK cell proliferation on inflammatory cytokines such as IL-15, IL-21, IL-18 and IL-12.

The ability of CD8 T cells, but not NK cells, to generate their own fuel, autocrine IL-2, which regulates effector T-cell metabolism, is another distinguishing factor. In cytotoxic CD8^+^ T cells, the activation and persistence of the mammalian target of rapamycin (mTOR) complex mTORC1 is mediated by IL-2, which regulates both metabolic and transcriptional pathways [35]. Consequently, mTORC1 activity is crucial in governing energy-producing metabolic functions in cytotoxic lymphocytes. NK cells rely on fuel in the form of cytokines, mainly IL-15 but potentially also IL-10, IL-21 or IL-2, to activate the mTOR pathway [36], and NK cells do not synthesize their own IL-15 but instead require it to be produced by activated myeloid cells (e.g., DCs), epithelial cells or even some tumor cells. Thus, there is a need to consider how NK cells respond to IL-15 and how tumor-localized production of IL-15 (or IL-2, IL-21, or IL-10) is affected by therapeutics and how this therapy can be customized to address the likely insufficient cytokine receptor agonism within the TME. As highlighted in a recent Nature Outlook article on targeting NK cells in cancer [37], NK cell immunotherapies benefit from enhancing the IL-15 receptor signaling axis by adding IL-15 to therapeutics and/or targeting the negative regulators of IL-15 receptor signaling to increase the response of NK cells to the limited amount of endogenous IL-15 within the TME.

### Training NK cells for modern warfare

However, the engagement of specific activating receptors can augment cytokine-mediated NK cell proliferation. For example, NKp30 stimulation via agonistic antibodies or recognition of a cognate ligand (B7-H6) on target cells can significantly increase human NK cell proliferation via mTOR activation in the presence of low concentrations of IL-12 even without IL-15 [38]. Indeed, two of the most well-characterized pathways that lead to rapid expansion of NK cells involve the combination of simultaneous cytokine receptor and activating receptor engagement. Shortly after cytomegalovirus infection in both mice and humans, NK cells rapidly expand in infected organs in response to viral peptide expression on infected cells, resulting in acute increases in proinflammatory cytokines derived from antigen-presenting cells (IL-12, IL-18, IFNα/β and IL-15) [39]. To this point, the pool of NK cells expressing activating receptors for viral ligands preferentially expands over those NK cells that do not. Furthermore, in vitro culture protocols for expanding high yields of primary human NK cells utilize allogeneic tumor cell lines such as K562 cells expressing ligands for the NK cell receptors NKp30, 41BB and IL-21R as feeder cells in combination with IL-2/IL-15-containing media, and the combination of these signals is required for optimal NK cell expansion [40].

Some next-generation NK cell engager approaches will evaluate the benefit of cotargeting both cytokine receptors and activating receptors with their engager design. The preclinical tetraspecific engagers against NKp46, CD16a (FcγRIIIa) and CD20 expressed on malignant B cells include an IL-2 receptor binding element [31]. A mutant IL-2 variant with reduced affinity for the CD25/IL-2Rα chain (which is expressed constitutively by Tregs but is also induced on activated T cells and NK cells) forms the fourth arm of this tetraspecific engager, with the goal of preserving the NK cell frequency and function while reducing target-mediated drug disposition and preferential Treg binding and activation. When injected into B-cell lymphoma-bearing SCID mice, the trispecific NKp46-CD16a-CD20 molecule fails to induce endogenous NK cell activation, with the NK cell frequency, CD69 expression and Ki67 expression being identical to those in vehicle-treated mice. In contrast, the tetraspecific molecules (IL-2v-NKp46-CD16a-CD20) resulted in a large increase in the systemic NK cell frequency and Ki67, IFNγ, granzyme B and CD69 expression. This finding is not surprising since the IL-2v moiety has a similar affinity for IL-2Rγ/β as wild-type IL-2 or IL-15 does, as determined by the induction of pSTAT5, and systemic IL-15 and IL-2 administration induce similar expansion and activation of NK cells. These data clearly demonstrate the need to strategize cytokine receptor signaling when designing biologics that target endogenous NK cells. These molecules need to be thoughtfully designed since systemic IL-2Rγ/β agonism is not well tolerated in humans [41–43].

As such, a necessary consideration for targeting multiple receptors simultaneously (i.e., cytokine receptor plus activating receptor) with the NK cell engager format is how the affinities of each targeting arm affect the biodistribution, pharmacokinetics and potential toxicity profiles of the molecule. This is especially important when cytokines such as IL-2 or IL-15, which have affinities for their trimeric (Kd~1 × 10–11 M) and dimeric (Kd~1 × 10–9 M) receptors, which can exceed the affinity of the targeting antibodies to the NK cell-activating receptors, are used [44]. Others have looked at this problem differently and combined NK cell engagers with off-the-shelf allogeneic NK cells to achieve tumor-targeting and simultaneously high frequencies of NK cells able to bind the engagers and contribute to tumor killing. Preclinical studies clearly demonstrate the merit of this approach when a tetravalent bispecific antibody (with two binding sites for each CD30 and CD16A) is preincubated with cytokine-activated cord blood-derived NK cells, and human clinical trials are currently evaluating this approach in patients with recurrent or refractory CD30^+^ Hodgkin or nonHodgkin lymphomas (NCT04074746) [45].

### Keep the NK cell Army fit and focused

IL-15 promotes NK cell activation, proliferation, and survival, and multiple NK cell engagers, including GTB-3550 and GTB-7550, have incorporated a wild-type IL-15 moiety into their design [46, 47]. These molecules target CD16 on NK cells and CD33 or CD19 on leukemia cells and are currently in preclinical evaluation. Owing to the potential limitations of CD33 as a target antigen in AML and concerns over antigen escape, additional IL-15-containing NK cell engagers for AML are also under preclinical evaluation, including a molecule with a humanized anti-CD16 heavy chain camelid single domain antibody and a single-chain variable fragment targeting human CLEC12A [48].

Targeting NK cell metabolism and fitness, for example, by enhancing IL-15 receptor signaling, should also help overcome some of the immunosuppressive pressures bestowed on NK cells within the tumor microenvironment. Advanced tumors harbor NK cells with distinct phenotypes, but the precise timing, underlying mechanisms, and functional significance of these NK cells remain largely unclear. The functional status of tumor-resident NK cells has been shown by us and others to be markedly compromised compared with that of NK cells from nonaffected tissues [49–51]. Thus, emerging NK cell immunotherapeutic approaches must acknowledge and attempt to reverse or prevent further NK cell dysfunction to increase their chances of efficacy. However, whether and to what extent tumor-resident NK cell immunosuppression/dysfunction/exhaustion can be reversed and the dominant pathways involved in this process remain unclear. This is an area of much needed research. The kinetics of NK cell dysfunction were recently addressed in preclinical tumor models via the very elegant technique of temporal labeling of tumors via photoconversion combined with longitudinal transcriptomic and ex vivo cellular analysis to track the fate of NK cells after they are recruited into the solid tumor microenvironment [50]. Single-cell RNA-seq of labeled NK cells revealed that NK cells rapidly lose their cytotoxic and inflammatory effector functions and develop a phenotype associated with tissue residency, including the expression of CXCR6, CD49a and the inhibitory receptors NKG2A, CD96 and LAG3. This phenomenon was originally described in a range of orthotopic preclinical solid tumors and referred to these tissue-resident NK cells as type 1 innate lymphoid cells (ILC1s) [49]. Conventional mature circulating NK cells were found to differentiate into ILC1s in tumors via an intermediate state termed intILC1s. Using genetic mouse models of NK cells with constitutively active TGF-β receptors or NK cells lacking the TGF-β receptor, it was shown that the tumor microenvironment-derived TGF-β was a potent inducer of NK cell tissue residency or the ILC1 phenotype and associated dysfunction [49]. A more recent study on these data revealed that PG2E and hypoxia were not major determinants of this NK cell residency conversion or the ILC1 state in tumors. They also reported that depleting NK cells from established tumors had no effect on tumor growth kinetics, suggesting that NK cells within large established tumors no longer efficiently contribute to antitumor immunity [50]. Consistent with our previous findings, the treatment of tumor-bearing mice with systemic IL-15 prevented NK cell dysfunction and enhanced their antitumor function, as determined by a retardation in tumor growth kinetics, although this effect was also partly driven by the survival and differentiation effects of IL-15 on CD8 + T cells. Importantly, the authors were able to validate their findings in human colorectal cancer biopsies where they identified a similar tumor-resident NK cell population with a tissue-resident phenotype and impaired effector molecule expression, highlighting the challenging environment for NK cells in solid tumors and the importance of addressing metabolism and dysfunction with NK cell immunotherapies.

### Navigating the TGF-β minefield

Understanding how the tumor microenvironment suppresses NK cell tumor immunity by inducing ILC1 differentiation and whether this process is reversible will open the door to novel approaches aimed at overcoming this phenomenon. To this end, it was recently shown that NK cell differentiation into ILC1s can occur in the absence of TGF-β and outside of a solid tumor microenvironment. When the role of the histone methyltransferase DOT1L in NK cell development was investigated, conditional *Dot1l* deletion at the immature NK cell stage was shown to result in the spontaneous generation of intILC1s in peripheral lymphoid organs characterized by the ectopic expression of CD49a [51]. Transcriptionally, these DOT1L-null NK cells closely resembled ILC1s and presented signs of immunosuppression with reduced expression of cytotoxicity genes compared with normal NK cells. The compromised immune set point of DOT1L-null NK cells was evident in their inability to expand in response to MCMV infection and their impaired cytotoxicity against various tumor cells. As mentioned above, NK cell differentiation into an ILC1-like state in solid tumors occurs rapidly after NK cell infiltration, and while most control NK cells acquire an intILC1 or ILC1 phenotype in orthotopic MC38 tumors, the proportion of ILC1 cells is greater in tumors of mice lacking DOT1L in their NK cells, which is correlated with poorer tumor control. These findings highlight the importance of H3K79 methylation in regulating NK cell lineage fate and preventing ILC1 differentiation and suggest that targeting histone methylation in NK cells could be explored to prevent immunosuppression driven by ILC1 differentiation. These data may also have implications for NK cell immunity in acute myeloid leukemia patients treated with DOT1L inhibitors [52].

The effect of TGF-β receptor signaling on the suppression of effector immune cell function is well established and further outlined above. TGF-β biology is complex and has beneficial and detrimental effects on both intrinsic and extrinsic tumor cells. A TGF-β-rich immunosuppressive TME can also promote cancer cell proliferation, angiogenesis, invasion, tumor progression, and metastasis [53]. Extensive preclinical research underscores the potential of targeting TGF-β as a promising strategy against solid advanced cancers. Several types of TGF-β inhibitors have emerged, including neutralizing antibodies, bifunctional antibodies, antisense oligonucleotides and kinase inhibitors. Despite encouraging results in preclinical models, translating these findings into clinical activity has proven challenging. Several recent trials in this space explored the potential of bintrafusp alfa (M7824), a bifunctional fusion protein comprising the extracellular domain of human TGF-β receptor II (TGF-βRII) linked via a flexible linker to the C-terminus of each heavy chain of an anti-PDL1 antibody [54–59]. In preclinical investigations, bintrafusp alfa significantly prolonged survival and induced durable protective immunity compared with either TGF-β or PDL1 blockade alone and markedly enhanced the infiltration of NK cells within tumors [59], although the ILC1/tissue residency differentiation status has not been reported. Some clinical trials of bintrafusp alfa are still active, although most have been terminated with late-phase trials, such as first-line treatment with bintrafusp alfa in patients with high PDL1 expression and advanced non–small-cell lung cancer, which fail to observe improved efficacy compared with anti-PD1 therapy (NCT03631706). Another ‘next-generation’ human anti-TGF-β monoclonal antibody (SAR439459) that inhibits all TGF-β isoforms and again demonstrated additive preclinical benefits to anti-PD1 therapy was also recently terminated. The results from a phase I/Ib first-in-human study of SAR439459 ± anti-PD1 in patients with advanced solid tumors (NCT03192345) revealed decreased TGF-β levels, modulation of peripheral NK cells and signs of conversion of tumor tissue from ‘cold’ to ‘hot’, although additional toxicity was observed [60–62].

## Cytokine Provisions. The NK Army Marches on its Stomach

**Fig. 3:**
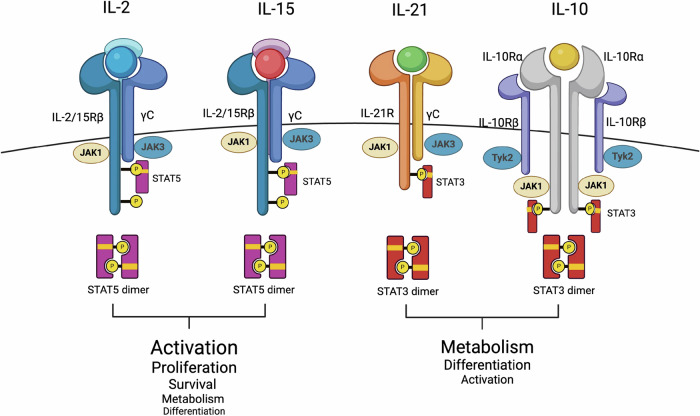
Cytokines and cytokine receptor signaling pathways relevant to NK cell antitumor immunity. Cytokines that preferentially activate STAT5 can enhance NK cell activation, proliferation and effector function within the tumor microenvironment. Cytokines targeting STAT3 can improve NK cell metabolism, potentiating NK cell effector responses and limiting exhaustion/dysfunction within the TME

A common theme in the preceding section on NK cell biology and cancer immunity is the importance of cytokines in maintaining NK cell survival, metabolism, and effector function and driving NK cell-dependent tumor immune infiltration (Fig. 3). Thus, it is not surprising that extensive immunotherapy drug development efforts are dedicated to novel cytokine-based immunotherapies. The use of cytokine drugs to enhance effector immune cell function and antitumor immunity is not new, with IL-2 and pegylated IFNα2b being FDA-approved in the late 1980s and early 1990s for the treatment of metastatic melanoma, renal cell carcinoma, AIDS-related Kaposi sarcoma and follicular lymphoma [41, 63]. At the time, advanced melanoma was considered incurable, with a median overall survival between 8 and 10 months. IL-2 was the first approved immunotherapy for stage IV melanoma, with a 1999 meta-analysis reporting a 15.9% objective response rate. Among the initial responders, 27.9% remained progression free at the time of reporting, with overall survival showing a plateau after 36 months. The nature of the metastatic disease, the short half-life of recombinant IL-2 and its preferential affinity for Tregs indicate that a high-dose bolus of intravenous IL-2 is needed, resulting in peripheral immune cell activation and systemic toxicity. These adverse events are common and severe, leading to a high incidence of grade 3/4 toxicities and requiring administration in an intensive care unit. Common adverse events include hypotension, which is attributed to capillary leak syndrome and resembles systemic inflammatory response syndrome [64].

Numerous additional cytokine therapies have been developed and tested over the past 30 years, yet dose-limiting systemic toxicities have been a common impediment in clinical development. Several key limitations of cytokine therapies exist. First, the poor pharmacokinetics (PK) of cytokines are due to target-mediated drug disposition (TMDD) or the ‘sink effect’ given the extremely high affinity of cytokines for their receptors on peripheral immune cells. Second, the very narrow therapeutic window between the severely toxic dose and therapeutic dose is due to the lack of tumor-targeting ability of the drug and, finally, tachyphylaxis or a reduced drug pharmacodynamic (PD) response upon subsequent dosing. These issues collectively work against the desired on-target (tumor/tumor-draining lymph nodes) PD of cytokine therapies, meaning that most of the drug is bound and cleared by cytokine receptors on nontarget peripheral immune cells in the blood and lymphoid tissues, resulting in very few cytokine drugs that affect the intended immune cell population. Only now, sophisticated engineering is the field making progress in addressing these issues, and this will be discussed in the following section.

### Cytokine provisions. IL-2

IL-2 binds the high-affinity trimeric IL-2Rα/β/γ expressed on Tregs and activated conventional and invariant T cells and the medium-affinity IL-2Rβ/γ on NK cells, invariant T cells, and tissue-resident memory (TRM) and conventional memory CD8 T-cell subsets [35, 44]. Depending on the level of IL-2R agonism, IL-2 promotes lymphocyte proliferation, survival, and metabolism and augments effector responses induced by activating receptors such as the T-cell receptor. While NK cell researchers originally used IL-2, not the physiologically relevant IL-15 (since it was discovered later) to study NK cell biology, IL-2 is not globally viewed as an appropriate cytokine for targeting NK cells in cancer because of its preferential binding and activity on Tregs and activated T-cell subsets. Thus, some improvements in regression-free survival resulting from high-dose IL-2 therapy may be mediated in part by improved NK cell frequency and antimetastatic immunity, as high-dose IL-2 treatment increased peripheral NK cell numbers in humans, nonhuman primates and mice and improved their ex vivo effector function [41, 65, 66]. Given the activity of IL-2 on a broad range of effector lymphocytes, its preferential activity for Tregs and its lack of specificity for targeting tumor-resident NK cells or neoantigen-specific T cells, most IL-2 drug development activity is focused on engineering cytokines to preferentially bind and act on activated conventional T cells, which in cancer patients are likely enriched in neoantigen-reactive T cells [67, 68]. Several engineered IL-2 variants (IL-2v) with attenuated CD25/IL2Rα binding have been tested in large clinical trials as monotherapies and combinations with anti-PD1 agents but have failed to improve the overall response rate at the maximum tolerated dose. One such innovation was Bempegaldesleukin, in which IL-2 was conjugated to six releasable polyethylene glycol (PEG) molecules located where IL-2 binds to CD25, thus impairing its binding to the trimeric receptor on Tregs. This approach increases tumor exposure in preclinical models by increasing the half-life and expanding the number of tumor-infiltrating CD8 + T cells and NK cells in murine models. Bempegaldesleukin monotherapy resulted in T-cell and NK cell proliferation and increased their accumulation in tumor biopsies after treatment compared with pretreatment [69–74]. Recent advances in our understanding of T-cell biology suggest that IL-2Rα/β/γ engagement by IL-2 on tumor-specific CD8 + T-cell subsets is essential for optimal CD8 + T-cell priming, “better effector” T-cell differentiation and antitumor immunity [75–77]. By upregulating CD25 upon activation, neoantigen-producing T cells should receive potent, preferential IL-2R signaling over nonantigen-specific T cells and NK cells. Flooding a system in which non-CD25-binding IL-2, such as Bempegaldesleukin, does not favor neoantigen-specific T cells and may impede their activity by expanding nonantigen-specific T-cell clones that compete for growth factors and nutrients. Indeed, in preclinical models, only wild-type IL-2, but not IL-2v, had additive effects when combined with anti-PD1 therapy to preferentially expand antigen-specific PD-1^+^ CD8^+^ T cells [75–77]. As such, biopharma revisits the design of IL-2-based drugs in an attempt to bias this potent IL-2R signaling toward neoantigen-specific T cells expressing PD1. Numerous examples of this approach exist where potency-attenuated IL-2 is fused to anti-PD1 (anti-PD1-IL2v) with the intention of restoring maximal IL-2R agonism on PD1^+^ T cells and minimal IL-2R agonism on PD1^−^ T cells. This concept of antibody-directed avidity is well established and can bias IL-2R signaling 100-fold on PD1^+^ versus PD1^−^ T cells [75–77]. In preclinical tumor and viral models, combining individual agents, such as IL-2v plus anti-PD1, was far less efficacious for disease control than single agents, such as the anti-PD1‒IL2v agent, highlighting the advantage of cis-targeting IL-2 on the right cell type in subsequent cytokine therapies. When combined with individual agents, IL-2v plus anti-PD1 is also less efficacious for disease control than is the full potency of IL-2 plus anti-PD1 because of the reduced binding of IL-2v to IL-2Rα [75–77]. Importantly, while many of the current anti-PD1-IL2v molecules use a ‘nonalpha’ IL-2v, if the molecule is dosed adequately and PD1 is sufficiently expressed on CD25^+^ T cells, then potent IL-2R agonism can be achieved on the desired target cell. Another consideration is that most Tregs also express high PD1 levels; thus, aPD1-IL2v will not discriminate between neoantigen-specific effectors and Tregs, meaning that aPD1-IL2v molecules would also promote Treg biology, including CD8^+^ T-cell suppression, and might be best used in combination with emerging Treg-depleting antibodies, such as anti-CCR8 [78, 79].

There are data suggesting that a minor fraction of tumor-resident NK cells express PD1 in humans and thus may also be targeted by the abovementioned approach [80]. In addition, some emerging NK cell engagers in development include an IL-2v moiety aimed at improving NK cell fitness and function [31]. This is likely an important addition to such biologics since NK cell activity and cytotoxicity rapidly decrease following tumor recognition and lysis, presenting issues if sustained NK cell serial killing is the desired outcome of the therapy. An additional benefit is that strong IL-2Rβ/γ agonism can also partially overcome immunosuppression caused by factors enriched in the TME, such as TGF-β [49, 81, 82]. However, it is important to acknowledge the divergent biological effects of IL-2 and IL-15 on NK cells and that caution should be taken when these two cytokines are used interchangeably. Despite their high homology and ability to bind to the same dimeric IL-2Rβ/γ, fully potent (wild-type) IL-2 and IL-15 induce distinct transcriptional signatures in T cells and NK cells owing to differing receptor binding kinetics impacting the magnitude, duration and breadth of JAK/STAT activity [83]. This ultimately impacts the spectrum of target genes activated by each of these cytokines in either NK cells or T cells. This needs to be kept in mind when nonattenuated or potency-enhanced IL-2 or IL-15 are used as therapeutics since these cytokines can also outcompete endogenous cytokines for their preferred receptors, impacting normal NK or T-cell biology. For example, an experimental therapeutic containing nonattenuated IL-2 could outcompete endogenous IL-15 for IL-2Rβ/γ on endogenous NK cells, skewing their homeostasis and function, and vice versa. However, if an engineering strategy could be designed to preferentially deliver IL-2Rβ/γ agonists to NK cells over T cells, especially in the setting of hematological malignancies, this strategy may have merits alone or in combination with NK cell engagers or targeted antibodies. Indeed, IL-2 has been shown to increase the expression of NKp30, a receptor strongly implicated in the control of certain hematological malignancies, on NK cells [84]. The safety of such approaches requires careful oversight, as it is likely that hyperactivity of peripheral NK cells contributes to severe adverse events in human cancer patients treated with high-dose IL-2.

### Cytokine provisions. IL-10

IL-10 has been well characterized as an immunosuppressive cytokine produced by Tregs, NK cells and B-cell subsets; however, IL-10 also has the potential to counteract immunosuppressive mechanisms. At lower doses, IL-10 has anti-inflammatory effects, whereas higher concentrations promote the activation and proliferation of tumor-infiltrating CD8^+^ T cells [85]. The IL-10 receptor is expressed on myeloid and lymphoid cells, including NK cells and CD8^+^ T cells, and IL-10 has been shown to increase their activation, proliferation and survival, suggesting that IL-10 could be exploited as an immunotherapy agent [86, 87]. As such, pegylated IL-10 (pegilodecakin) was developed and extensively tested for cancer therapy [88]. Pegilodecakin enhances the cytotoxicity and proliferation of IL-10R^+^ CD8^+^ T cells, and in preclinical models, pegilodecakin enhances the frequency of CD8^+^ T cells, promoting tumor immunity [89]. Furthermore, early clinical trials reported monotherapy objective responses in 4 of 15 renal cell carcinoma patients without severe toxicity [90, 91]; however, the clinical development of IL-10 therapies has not progressed further in oncology. IL-10 induces similar antitumor effector functions in NK cells, including improved cytotoxicity and protection from activation-induced cell death, making it an attractive target if it can be preferentially delivered to NK cells [92, 93]. NK cells also rapidly express IL-10 during acute infection, which was shown to be dependent on IL-12 [94, 95]. Given the known role of IL-12 in NK cell activation during tumor immunity, it is possible that IL-10 produced by NK cells can act in an autocrine fashion to increase or maintain NK cell activity. However, given that IL-10 has preferential affinity for myeloid cells due to its increased expression of IL-10R, it is likely that NK cell-derived IL-10 could also drive immunosuppression to shut off an immune response to pathogens or within the tumor microenvironment [85]. Indeed, NK cell-derived IL-10 was shown to suppress IL-12 production from DCs during systemic pathogen infection [96, 97]. The potency of attenuated IL-10-Fc drugs is now being investigated for the treatment of autoimmune and inflammatory diseases.

### Cytokine provisions. IL-15

In the 2008 summary of the National Cancer Institute Immunotherapy Agent Workshop titled “Twelve immunotherapy drugs that could cure cancers”, Mac Cheever wrote in his article that if tested creatively and in combination, immunotherapy regimens will be derived to benefit many cancer patients, and IL-15 was ranked as the most promising candidate. Since then, significant efforts have been made to develop IL-15 as a therapeutic agent. Indeed, IL-15 is very similar to the FDA-approved IL-2 but does not preferentially engage CD25 and expand Tregs, much like the more recent IL-2v molecules highlighted above [43, 98]. However, systemic delivery of IL-15 has failed to progress through clinical development because of dose-limiting toxicity, and the future of IL-15 therapies will require innovations such as potency attenuation and antibody targeting or masking/tumor conditional activation to reduce systemic activity and direct IL-15R agonism to effector cells at the site of disease [99–101]. An alternative but very narrow application is the intratumoral administration of IL-15. Recently, IL-15 received its first FDA approval in the form of an IL-15R superagonist (Anktiva/N803/ALT803) administered intravesically in combination with Bacillus Calmette-Guerin (BCG) for the treatment of patients with BCG-unresponsive non–muscle-invasive bladder cancer [102–104]. For most cancer patients, the intratumoral administration of immunotherapy is not an option; thus, more work is needed to improve the safety and targeting of IL-15 agents; however, this recent approval highlights the transformative potential of IL-15R agonists if they are specifically targeted to the TME.

The cis-cytokine signaling approach mentioned above for IL-2 is also being applied to IL-15. Several antibody‒cytokine fusion molecules linking anti-PD-1 with potent attenuated IL-15 (IL15v) are in development, including SAR445877 and PF-07209960. This immunocytokine is designed to restore the potency of IL-15R agonism preferentially to PD-1^+^ lymphocytes via avidity. The anti-PD1 agent used is a functional blocking antibody, and the combination of IL-15R agonism and PD-1 blockade in the same cell has the desired effect; however, only low doses of these current anti-PD-1-IL-15v-Fc agents are tolerated, indicating that PD-1 receptor occupancy is insufficient to block PD-1 signaling [105]. Potency attenuation aims to minimally stimulate circulating peripheral NK cells, as it has been found to drive the dose-limiting toxicity associated with anti-PD1-IL15v-Fc [106–108]. SAR445877 contains a single amino acid mutation conferring reduced potency of IL-15Rβγ stimulation relative to that of wild-type IL-15, with reduced binding to IL-2Rβ and decreased pSTAT5- and IL-15-dependent proliferation. In preclinical tumor models, this approach has demonstrated strong antitumor effects, outperforming both single agent IL-15 superagonist and single agent anti-PD1 therapy, as well as their combination in melanoma models (AACR poster 2023). Single-cell RNA sequencing revealed that this treatment promoted the expansion of a cluster of exhausted CD8^+^ TILs with high proliferative capacity and effector-like characteristics. Whether the same approach can be used to preferentially target tumor-resident NK cells or combine with NK cells to generate biologics warrants further investigation. Another next-generation immunocytokine approach is the tumor-conditional prodrug format, meaning that the molecules are designed to become functional predominantly in the tumor microenvironment. A very recent example is LH05, an anti-PD-L1-IL-15 prodrug where IL-15 is masked through steric hindrance by the antibody FC domain, reducing the “cytokine sink” effect driven by the high affinity for IL-15R and minimizing the associated systemic toxicity [108]. The molecule contains urokinase-type plasminogen activator (uPA) proteolytic cleavage sites at the junction of IL-15 and anti-PD1; thus, within the tumor microenvironment (often enriched with uPA), LH05 is cleaved, resulting in local release of the IL-15R agonist and remarkable antitumor immunity, even in cold tumors. An issue with conditional activation approaches for cytokine drugs continues to be the peripheral activation and insufficient difference in the magnitude of cleavage or unmasking in the TME versus the periphery.

In addition to the reduced potency of immunocytokines and the local delivery and tumor conditional activation of IL-15, the identification and targeting of IL-15 signaling checkpoints is also gaining interest as an approach to increase the therapeutic index of targeting IL-15R. Inhibitory T-cell immune checkpoints such as PD1 are induced in response to potent T-cell receptor stimulation to prevent overt immune activation, immunopathology and autoimmunity. Similarly, cytokine checkpoints are induced in immune cells following strong engagement of cytokine receptors, such as during viral infections and some cancers [98]. IL-15Rβ/γ crosslinking on NK cells results in the activation of JAK1/3 and the preferential induction of pSTAT5 over pSTAT3. The cytokine-inducible SH2-containing protein-encoding gene *CISH* is a STAT5 target gene that is induced in response to IL-15R signaling. The encoded protein, CIS, binds to phosphotyrosine residues in the IL-15R and JAK1/3 proteins via its SH2 domain and may compete for STAT5 binding sites to limit STAT5 activity [109]. Furthermore, CIS contains a SOCS-BOX domain that can recruit the EloginB/C-CUL5 ubiquitin-ligase complex to mediate substrate degradation and cessation of signaling. Importantly, CIS mediates the downregulation of IL-15R on NK cells, with small changes in the levels of IL-15R/CD122 having a large effect on the NK cell response to physiological levels of IL-15 [109]. In *Cish*^*−/−*^ mice, NK cells at steady state express slightly elevated IL-15R/CD122 levels, which results in a significant increase in NK cell sensing of IL-15, as evidenced by increased Ki67 levels in *Cish*^*−/−*^ NK cells. Furthermore, *Cish*^*-/-*^ NK cells are somewhat resistant to the downregulation of IL-15R in the presence of IL-15, meaning that they maintain elevated and prolonged JAK/STAT signaling at sites of inflammation, including tumors, resulting in dramatic improvements in NK cell effector function and tumor immunity [81, 109, 110]. The sensitivity of NK cell biology to small changes in CD122 levels was also recently revealed in mice in which *Ikzf1* is conditionally deleted in NK cells. *Ikzf1-*null NK cell development is significantly impaired due to a 10–15% downregulation of IL-15RB/CD122 on NK cells in the steady state since IKZF1 binds directly to *Cish*, repressing its transcription. As a result, *Ikzf1-*null NK cells have elevated *Cish* expression, reduced CD122 surface expression and weaker IL-15R signaling [111]. This biology is well conserved in human NK cells, with the deletion of *CISH* promoting IL-15R signaling and improving antitumor function, particularly in the context of CAR-NK cell antileukemic immunity in vivo [112, 113]. Targeting the negative regulators of IL-15R signaling is an attractive approach, as it opens the door to designing small molecules that inhibit enzymes involved in cytokine receptor expression and signaling, akin to the recent small molecules in cancer trials that target PTPN1B/2 and promote IFNγR signaling among other receptors [114, 115].

### Cytokine Provisions. IL-21

IL-21 is another common gamma chain (γC) cytokine family member implicated in antitumor immunity [116]. IL-21 is derived from activated T and NKT cells and has an established role in B and T-cell differentiation. IL-21 signals through a unique form of IL-21Rα via JAK1/3, preferentially recruiting and activating STAT3 and, to a lesser extent, STAT1, as opposed to the dominant activation of STAT5 by other γC cytokines, such as IL-2 and IL-15. In isolation, IL-21 does not promote NK cell proliferation, and when NK cells are cultured with both IL-15 and IL-21, IL-15-induced proliferation is retarded, and NK cell activation and differentiation are enhanced with increases in cell size, cytotoxicity and cytokine production (IFNγ, CCL3/4 and specifically IL-10). Paradoxically, both human and mouse NK cells exhibit dramatic upregulation of CD94/NKG2A receptor dimers in response to IL-21. The CD94/NKG2A inhibitory receptor complex can bind to HLA-E (Qa-1b in mice) and transmit inhibitory signals to NK cells, with CD94/NKG2A typically being lost during NK cell maturation/differentiation, as NK cells acquire other inhibitory receptors, such as Ly49/KIRs and KLRG1.

Recently, the therapeutic potential of IL-21 was further exemplified by the group of Prof. Rezvani, who engineered cord blood-derived NK cells to express and secrete autocrine IL-15 or IL-21 [117]. Compared with IL-15-expressing NK cells, IL-21-producing NK cells presented superior activation and proliferation; increased ATP production through oxidative phosphorylation (indicating increased metabolic efficiency); and increased expression of Ki67, CD25, CD95/FAS, IL-10, IFNγ, CCL3, CCL4, TNFα, Granzyme B and perforin. Importantly, these parameters were measured in NK cells cocultured with a glioblastoma cell line that was sensitive to NK cell lysis, and tumor-ligand binding is likely a key determinant of enhanced NK cell proliferation in the presence of IL-21. Indeed, as mentioned earlier, large-scale manufacturing batches of human NK cells for clinical use commonly use feeder cells (typically a tumor cell line such as K-562) expressing IL-21 and activating ligands. Unlike IL-15-expressing NK cells, which are translated into significant reductions in disease burden in preclinical models of glioblastoma, IL-21-expressing NK cells potently kill tumor cells. Compared with IL-15, IL-21 induced distinct epigenetic and transcriptional changes in this study, including the activation of the C/EBP, IKZF, and AP-1 transcription factor families, with STAT3 activation by IL-21R increasing CEBPD expression, and was found to be essential for NK cell proliferation and antitumor activity. IL-21-based biologics are still being investigated preclinically, with an anti-PD1-IL-21-mutein molecule showing antitumor activity in preclinical models [116, 118] and an IL-7/15/21R agonist (HCW9201) being investigated to expand NK cells in vivo [119]. Phase I/II trials of recombinant IL-21 revealed that it was generally well tolerated with dose-limiting toxicity, including increased transaminase levels, hyperbilirubinemia, hypersensitivity reactions, and lethargy. Increased biomarkers of cytotoxic immune cell activation, including increased soluble CD25, GZMB^+^ CD8^+^ T cells and NK cells, were observed. Clinical activity has been noted in patients with multiple myeloma, renal cell carcinoma and advanced melanoma [120–126].

## Revising our NK cell Strategy in the War on Cancer

In this review, we highlight the numerous direct and indirect antitumor properties of NK cells and some of the past and ongoing attempts to harness these properties therapeutically. The challenge for the NK cell drug discovery field is how to design therapeutic approaches that safely and persistently target endogenous NK cells for durable antitumor activity in advanced disease settings. Successful drug development requires a clinical signal (improvement in disease status while on investigational agent therapy) over a relatively short period (several months) depending on the stage of the disease being treated. NK cell activity is associated with improved progression-free survival and subsequently overall survival, likely owing to the role of NK cells in immunosurveillance of metastases; however, long-term trials accessing investigational agent activity in preventing disease relapse are logistically and strategically difficult trials to run, as relapse can take many years to occur depending on the disease. Thus, NK cell drug development will need to focus on approaches that target NK cells to orchestrate a more potent, rapid and durable endogenous immune response to cancer that can be read out in advanced, progressive cancer types over the short term. Clinical trials involving novel agents harnessing endogenous T-cell anticancer immunity have yielded several FDA approvals over the past 10 years, but NK cell drug developers need to be cognizant of the mechanisms by which endogenous T cells are targeted and how they differ from NK cell biology. Elucidation of the dominant pathways that activate tumor-resident NK cells and the development of drugs to do the same are likely to have a transient response only if no consideration is given to how these drugs might negatively impact the persistence (metabolic fitness, survival, homeostasis, proliferation, and activation-induced cell death) of tumor-resident NK cells. Several approved therapies targeting T cells exploit the ability of these agents to not only activate, restore or redirect T-cell activity toward tumor cells directly but also drive T-cell proliferation, increasing the drug target and magnitude and duration of the drug response. Novel NK cell-targeting approaches include modalities to prevent or minimize the negative impacts that strong NK cell activation has on NK cell viability and function or to target qualitatively different pathways that preserve NK cell persistence during antitumor immunity.

Our understanding of T-cell biology dwarfs that of natural killer (NK) cell biology, but through our increased basic and translational NK cell research endeavors, the substantial insights that functional genomic NK cell/tumor cell screens offer, as well as the ever-expanding repositories of scRNA-seq, bulk RNA-seq and proteomics data from healthy and disease tissues, we are better equipped than ever to design, develop and test the next generation of natural killer (NK) cell-targeting agents in oncology.
